# Perception Regarding Early Clinical Exposure among Second Year Medical Students after Educating Pregnant Women on Physiological Changes during Pregnancy: A Mixed Methods Study

**DOI:** 10.31729/jnma.7289

**Published:** 2022-02-28

**Authors:** Bipin Kumar Shrestha, Bikalp Thapa, Rashmi Shrestha, Tara Man Amatya, Ratna Khatri

**Affiliations:** 1 Department of Physiology, Nepalese Army Institute of Health Sciences, Kathmandu, Nepal; 2 Department of Pharmacology, Nepalese Army Institute of Health Sciences, Kathmandu, Nepal; 3 Department of Gynecology and Obstetrics, Shree Birendra Hospital, Kathmandu, Nepal

**Keywords:** *academic performance*, *early clinical exposure*, *perception*, *physiology of pregnancy*

## Abstract

**Introduction:**

The traditional teaching-learning process should reform to improve the academic performance and understanding of the students. This study aimed to determine the perceptions of second-year medical students towards early clinical exposure about their approach to educating pregnant women on the physiology of pregnancy.

**Methods:**

This was a descriptive cross-sectional study with a mixed-method design comprising both quantitative and qualitative components among second-year medical students of a medical college in Nepal from September 2019 to September 2020. After ethical approval from the Institutional Review Committee (Reference number: 207), 40 students included through the convenience sampling method. These students were subjected to early clinical exposure in the form of educating pregnant women on physiological changes during pregnancy. Data was entered and analyzed using Microsoft Excel 2016. Point estimate at 95% Confidence Interval was calculated along with frequency and proportion for binary data.

**Results:**

Among 34 responses, majority of the students 29 (85.28%) (73.36-97.20 at 95% Confidence Interval) were motivated to learn the physiology of pregnancy after the activity; 15 (44.11%) strongly agreed and 14 (41.17%) agreed to this statement. Thirty-two students (94.11%) claimed that the activity improved their understanding of the physiology of pregnancy. The majority of the students expressed that this approach is pragmatic which ignited more curiosity regarding the subject matter.

**Conclusions:**

The majority of the students had satisfactory perceptions regarding their early clinical exposure which was similar to standard data and they expressed that they would like to have similar activities in the future.

## INTRODUCTION

Educational innovation is required for active teaching-learning strategies and teachers need to be more innovative in active learning classes.^1-3^ In certain parts of the world, classroom-based didactic lectures still account for the majority of teaching-learning methods.^4-8^ The students prepare theory-based answers to pass exams that do not adequately meet the goal that is patient service. Hence, lecture-based learning during the first two years in medical school is not enough for the students to contextualize the basic science concepts clinically.^6,7^

The passive lecture style is tedious and monotonous.^9^ Students who are actively engaged in learning hold knowledge for longer periods than the passive receivers of guidance.^10^ Compared to traditional lectures, active learning strategies are preferred by the students.^11^ Students' understanding of the subject and their retention improve from active participation.^1^

This study aimed to determine the perceptions of second-year medical students towards early clinical exposure about their approach to educating pregnant women on the physiology of pregnancy.

## METHODS

This was a descriptive cross-sectional study with a mixed-method design having both quantitative and qualitative components. This study was conducted among second-year medical students of the Nepalese Army Institute of Health Sciences - College of Medicine from March 2019 to September 2020. The ethical approval from the Institutional Review Committee-NAIHS was obtained with reference number 207.

For both quantitative and qualitative components, the study population included second-year medical students of a medical college.

### QUANTITATIVE COMPONENT

The study population included a sample of 40 students who were recruited to be part of the study through the convenience sampling method.

The sample size was calculated using the formula:

n = Z^2^ × p × q / e^2^

  = (1.96)^2^ × 0.5 × (1-0.5) / (0.1)^2^

  = 96

Where,

n = minimum required sample size
Z = 1.96 at 95% Confidence Interval (CI)
p = prevalence taken as 50% for maximum sample size
q = 1-p
e = margin of error, 10%

For the correction for finite population, using the formula

  n= n / (1 + n / N)

  = 96 / (1 + 96 / 55)

  = 35

Where,

n = calculated minimum required sample size, 96
N = total number of second-year medical students, 55

Hence, after adjustment for a finite population, the sample size required became 35. To this, a 10% nonresponse rate was added giving a sample size of 39. However, we took a sample of 40 students in our study.

Data was entered and analyzed using Microsoft Excel 2016. Point estimate at 95% Confidence Interval was calculated along with frequency and proportion for binary data.

### QUALITATIVE COMPONENT

Firstly, the students were subjected to four lectures (180 min) on reproductive physiology according to the medical course curriculum with the following objectives (Table 1) which were prepared and taken by the same teacher on four different days in a week. Then, a pretest consisting of multiple choice and true/false questions was completed by the students. Afterward, the students were divided into 8 groups (n= 5 students per group). Each group consisted of two girls and three boys who were randomly allocated. They were given one week to prepare the educational tools using native language on physiological changes during pregnancy. These consisted of flip charts, pamphlets, mannikins, models, diagrams, or any other innovative tools. The students were free to use the lecture notes for this. In the following weeks, each group (2 groups per week) went to visit ANC OPD in the institution's hospital for observing the clinical examination by the Obstetrician. During the hospital visit, the students educated the pregnant women using various tools that they had prepared while the pregnant women were waiting for their appointment. After 4 weeks all the groups had completed their activities. Lastly, all the students completed a posttest after the activity.

**Table 1:** Description of topics covered during lectures in Physiology of pregnancy.

Course objectives as per curriculum	Topic descriptions
a. The students should be able to outline the physiological changes during pregnancy	Hormonal and reproductive organs changes(Changes in estrogen and progesterone, the uterus, breast, cervix, and vagina) Pregnancy-related changes in posture and joints Changes in body weight during pregnancy Changes in the cardiovascular system (The heart, blood volume, cardiac output, blood pressure and edema during pregnancy) Respiratory system changes in pregnancy Gastrointestinal tract system changes in pregnancy Urinary system changes in pregnancy Changes in skin during pregnancy
b. The students should be able to explain the physiological basis of changes during pregnancy	Describe the physiological basis of the above changes

On the day the students completed their hospital activity, they were independently and anonymously asked four questions about their interactions with the pregnant women and the benefits perceived. Students were asked to respond to the questions using a Likert-type scale (from strongly agree to strongly disagree).

Perceptions of the students regarding the study were taken in the form of feedback. Among 40 students, six students were absent during the posttest day, and hence, 34 students filled out the feedback form. The feedback from the students was obtained on the basis of the following open-ended question:

**"Briefly mention what was the best thing regarding the visit to ANC OPD and what are the things that can be improved?"**

Written informed consent was taken from the students and the pregnant women and the responses were recorded. The data was collected in the form of prepost test scores and feedback from students. The data were entered in Microsoft Excel and later analyzed.

## RESULTS

### A. QUANTITATIVE

Out of 40 study participants, six (two girls and four boys) were absent during the posttest and were excluded from the study. Thirty-four responses were recorded. Out of these responses, 29 (85.28%) (73.36-97.20 at 95% Confidence Interval) students were positive about the motivation to learn the physiology of pregnancy after the activity. Among them, 15 (44.11%) strongly agreed and 14 (41.17%) agreed to this statement. The results showed that 32 (94.2%) of the students were eager to have such activities in the future. Thirty-two students (94.11%) claimed that the activity improved their understanding of the physiology of pregnancy. Twenty-eight (82.34%) students said they were better able to correlate the physiology of pregnancy clinically after the activity (Table 2).

**Table 2:** Perceptions of the students regarding the physiology of pregnancy after the activity (n= 34).

Questions/Characteristics	Category	Responses n (%)
Would you like to have similar activities in the future?	Yes No	32 (94.2) 2 (5.8)
The activity improved my understanding of the physiology of pregnancy.	Strongly disagree Disagree Neither agree nor disagree Agree Strongly agree	1 (2.9) 1 (2.9) 12 (35.29) 20 (58.82)
I was able to better correlate my knowledge of the physiology of pregnancy clinically after the activity.	Strongly disagree Disagree Neither agree nor disagree Agree Strongly agree	1 (2.9) 1 (2.9) 4 (12.5) 20 (58.82) 8 (23.52)
I was motivated and interested to learn the physiology of pregnancy after the activity.	Strongly disagree Disagree Neither agree nor disagree Agree Strongly agree	2 (5.8) 3 (8.8) 14 (41.17) 15 (44.11)

Thirty-four students consisting of girls 14 (41.17%) and boys 20 (58.82%) participated in the activity with an average age of 20 years. The results of the mean score of the students before and after the activity are as follows (Table 3).

**Table 3:** Mean score of students before and after the activity

Assessment description	Mean score of students before activity (Mean±SD)	Mean score of students after activity (Mean±SD)
MCQs	16±2.11	17.11±1.68
True/False	14.17±2.28	15.05±1.82
Total	30.17±3.57	32.26±2.50

### B. QUALITATIVE

Following this exercise, feedback was gathered from students about their experiences with this unique teaching-learning strategy. In this manner, input was divided into their experience and perspective, as well as areas for improvement.

**Feedback 1. Perception and experience:**

Best things (common to greater than 20 participants) regarding the visit to ANC OPD and educating the pregnant women on the physiology of pregnancy.

1. *"An opportunity to see in real what was read in the books"*
2. *"First exposure to the clinical environment. Felt like a doctor"*
3. *Increased curiosity about the subject matter*
4. *Listening to the fetal heart sound"*
5. *Learning while teaching*

**Feedback 2. Areas for improvement:**

1. *"Separate room/hall to explain to the patients with the use of audiovisual aids"*
2. *"More time and frequent visits to the hospital in relevant departments during the preclinical years"*

## DISCUSSION

Early Clinical Exposure (ECE) is defined as authentic human contact in a social or clinical context during the preclinical years (before the official clerkship and internship training programs).^13^ The advantages of ECE compared to traditional lecture-based learning are motivation and satisfaction in learning, better doctor-patient relationship, improved performance in exams, early understanding of the clinical environment. All this has been reiterated by many studies.^13,14^ Other benefits include improved communication skills, exposure to the health care system, instilling the qualities of a patient-centered humanistic physician, and increased motivation for learning.^15-17^

The advantages of using active learning techniques in education have been documented.^18-20^ As Michael J, et al. after reviewing the available data mentions that the feasibility of the proposed reforms, which include the use of student-centered, active learning pedagogy, is now well informed by evidence.^21^ Students who learn in this manner are more likely to achieve meaningful learning.^22^ The reviewer points out that individuals are more likely to learn with others than alone, and meaningful learning is encouraged by articulating explanations, whether to oneself, peers, or teachers.^21^ Here, the students were articulating explanations to the pregnant women.

The results of the present work show that involving students in educating pregnant women during ECE was a good strategy for teaching and learning the physiology of pregnancy for second-year students. It was evidenced by the result of our study that the majority (85.28%) of the students were motivated to learn the physiology of pregnancy after the activity. Similarly, 94.11% claimed that this methodology improved their understanding of reproductive physiology. This was evidenced by the improvement in the test scores after performing the activity. This, along with other active teaching methods, will improve student engagement and information retention, encourage student participation, and facilitate problem-solving.^23-26^

There are always tensions associated with innovation and the introduction of new pedagogical practices in various fields, necessitating careful evaluation of the evidence for the innovation's benefits in terms of available resources and preparation for change. The ability to resolve conflicts depends on striking a balance between the local context and the strategy's potential benefits.^27^ The findings, in this case, showed that implementing an active approach in conjunction with traditional lectures did not result in significant tensions among students or teachers. However, there was a bit of struggle in coordinating with the Department of Gynecology and Obstetrics and scheduling the students to the ANC OPD and some preceptors had difficulty changing their existing culture of clinical teaching. They were convinced by explaining the purpose of the study. There were positive interactions among the students when they were working on preparing education resources and educating the pregnant women in their groups. They seemed to enjoy the exercise. This was significant because students' output can be increased when the learning process is linked to a sense of enjoyment.^28-30^

When students take an active role in the learning process, they become more involved and interested in the process.^31^ Also, they take responsibility for the learning of others and become more creative.^18^ Students and lecturers working together as collaborators is a multifaceted process that involves the development of medical knowledge, skills, and attitude.^32^ Following this logic, the findings revealed that the majority of the students had an uplifting impression of the activity. Furthermore, the student's performance in the reproductive physiology tests increased as a result of the group activity.

The encouraging feedback from the students highlights the importance of such innovative teaching-learning methods not only to the students but also for pregnant women as they received useful information which may assist in the management or at least better understanding of their clinical conditions. This may apply to patients in other clinical situations. Similarly, in the review by Vijn TW, et al. various studies highlighted the impact of patient education by students integrated into medical education has the ability to increase treatment quality and medical education. Student-run patient education clinics, student-led outreach projects, student health coaching, and patient education clerkships, in particular, can improve care quality and medical education and should be incorporated into practice.^33^

Lastly, even though this sort of teaching-learning method is constructive to the students, it is both time and resource-intensive to the institute. Lots of coordination with the clinical department (Department of Gynecology and Obstetrics) was required for the smooth conduction of the project. Nevertheless, the benefits to the students outweigh the level of burden to the institute. Hence, after the results of the study were presented and explained with the institution authorities, they were convinced to implement this for the forthcoming batches.

The main limitation of this study was the small sample size of students. The pre-post tests were designed to be of a similar difficulty level, based solely on the author's judgment.

## CONCLUSIONS

Majority of the students had satisfactory perceptions regarding the early clinical exposure which was similar to standard data and would like to have similar activities in the future. Early Clinical Exposure in ANC OPD supplemented with educating pregnant women about physiological changes during pregnancy by the second-year medical students helped better understanding of the physiology of pregnancy, improved their test performance, increased the motivation, and made their learning more real and relevant. All the participant students believed that this was an interesting way of learning and contributed to their better understanding of the subject matter. In addition, most of the students requested more of such learning experiences. Such a teaching-learning approach should be incorporated into the medical curriculum due to its practical implications.
